# Application of a localized morphometrics approach to imaging-derived brain phenotypes for genotype-phenotype associations in pediatric mental health and neurodevelopmental disorders

**DOI:** 10.3389/fdata.2024.1429910

**Published:** 2024-12-11

**Authors:** Gabrielle Dagasso, Matthias Wilms, Sarah J. MacEachern, Nils D. Forkert

**Affiliations:** ^1^Department of Radiology, Cumming School of Medicine, University of Calgary, Calgary, AB, Canada; ^2^Hotchkiss Brain Institute, University of Calgary, Calgary, AB, Canada; ^3^Biomedical Engineering Graduate Program, University of Calgary, Calgary, AB, Canada; ^4^Alberta Children's Hospital Research Institute, University of Calgary, Calgary, AB, Canada; ^5^Department of Pediatrics, Cumming School of Medicine, University of Calgary, Calgary, AB, Canada; ^6^Department of Community Health Sciences, Cumming School of Medicine, University of Calgary, Calgary, AB, Canada; ^7^Department of Clinical Neurosciences, Cumming School of Medicine, University of Calgary, Calgary, AB, Canada

**Keywords:** imaging genetics, GWAS, neurodevelopmental disorders, principal component analysis, localized dimensionality reduction

## Abstract

**Introduction:**

Quantitative global or regional brain imaging measurements, known as imaging-specific or -derived phenotypes (IDPs), are commonly used in genotype-phenotype association studies to explore the genomic architecture of the brain and how it may be affected by neurological diseases (e.g., Alzheimer's disease), mental health (e.g., depression), and neurodevelopmental disorders (e.g., attention-deficit hyperactivity disorder [ADHD]). For this purpose, medical images have been used as IDPs using a voxel-wise or global approach via principal component analysis. However, these methods have limitations related to multiple testing or the inability to isolate high variation regions, respectively.

**Methods:**

To address these limitations, this study investigates a localized, principal component analysis-like approach for dimensionality reduction of cross-sectional T1-weighted MRI datasets utilizing diffeomorphic morphometry. This approach can reduce the dimensionality of images while preserving spatial information and enables the inclusion of spatial locality in the analysis. In doing so, this method can be used to explore morphometric brain changes across specific components and spatial scales of interest and to identify associations with genome regions in a multivariate genome-wide association study. For a first clinical feasibility study, this method was applied to data from the Adolescent Brain Cognitive Development (ABCD) study, including adolescents with ADHD (n = 1,359), obsessive-compulsive disorder (n = 1,752), and depression (n = 1,766).

**Results:**

Meaningful associations of specific morphometric features with genome regions were identified with the data and corresponded to previous found brain regions in the respective mental health and neurodevelopmental disorder cohorts.

**Discussion:**

In summary, the localized, principal component analysis-like approach can reduce the dimensionality of medical images while still being able to identify meaningful local brain region alterations that are associated with genomic markers across multiple scales. The proposed method can be applied to various image types and can be easily integrated in many genotype-phenotype association study setups.

## 1 Introduction

The integration of high-resolution brain imaging and genetic analysis has opened new avenues for understanding the complex interplay between neural structure/function and genetic predispositions in neurodevelopmental and psychiatric disorders. A contributing factor for this may be that data from these modalities are typically analyzed independently from each other, whereas imaging and genomic markers are inherently linked such that both data sources may only unlock their true potential when analyzed together. For this reason, genotype-phenotype analysis methods such as genome-wide association studies (GWAS), in which the phenotype includes brain neuroimaging data, may offer new avenues to explore the genomic architecture of the human brain and how it may be affected or altered in case of neurological and mental disorders. This may ultimately lead to novel knowledge and biomarkers that improve clinical diagnosis or result in new treatment options (Mascarell Maričić et al., 2020; Dagasso et al., 2020).

The genotypic component of these analyses commonly utilizes single-nucleotide polymorphisms (SNPs), which are single base position changes in an individual's DNA, to identify relevant differences between individual people or between groups at a population level (Gray et al., 2000). Analyzing these patterns can help to identify uncommon or even rare variants, which may contribute to a disease of interest. However, it is important to note that many diseases cannot be traced to a single SNP, but multiple SNPs that have a combined effect, which makes the identification of relevant SNPs challenging, given the vast number of SNPs that can be measured.

On the phenotypic side, genotype-phenotype studies typically use categorical information, such as disease status. However, such a discretization is often problematic, especially in case of neurological, mental health, and neurodevelopmental disorders that exist on a spectrum or consist of multiple sub-types. In those cases, indirect representations or endophenotypes (Elliott et al., 2018) derived from imaging modalities such as magnetic resonance imaging (MRI) may provide more information and benefit imaging genetics for detecting novel biomarkers (Saykin et al., 2010; Klein et al., 2019; Thompson et al., 2020). While using images directly as the phenotype may be theoretically beneficial, incorporating them into genotype-phenotype analyses is practically challenging due to the high dimensionality of such scans that may contain millions of voxels. One solution to this problem is to focus only on pre-selected regional imaging-specific or -derived phenotypes (IDPs) associated with specific brain structures to reduce computational burden (Narr et al., 2009). However, this may result in the loss of important localized information.

Including whole medical images, particularly T1-weighted MRI datasets due to their wide-spread use, as part of the phenotypic association within a GWAS is not a new concept and several studies have used voxel-wise testing for this purpose (Stein et al., 2010). For example, Rodrigue et al. (2020) used source-based morphometry, a neuroimaging methodology to describe volumetric changes on a voxel-by-voxel basis, using BGENIE (Bycroft et al., 2017), a linear approach for multiple-trait testing in a GWAS that was specifically designed for UK Biobank data (Bycroft et al., 2018). While multivariate testing methods like this can help to mitigate some multiple testing constraints related to the high dimensionality of images, dimensionality reduction is typically needed to allow for more spatially smooth results in comparison to potentially noisy results and to avoid other issues with voxel-wise testing, like computational time complexity.

One alternative to voxel-wise testing is to perform dimensionality reduction of the T1-weighted MRI datasets or other sequences using principal component analysis (PCA) prior to conducting a GWAS. However, this results in a spatially global description of the variation in the medical images. Such a global approach has, for example, been previously used to investigate associations with canonical component analysis (Mihalik et al., 2022) or sub-groups in disorders by non-negative matrix factorization (Anderson et al., 2014; Arnedo et al., 2015). Thus, instead of performing spatially highly localized genotype-phenotype association testing using the voxel-wise data, one is now modeling the data's global variability in far fewer dimensions. While theoretically sound, a potential issue with using PCA to reduce dimensionality of medical images is that multiple brain regions, which are not functionally or anatomically related or close in distance, may appear within one component. This directly leads to difficulties drawing conclusions about specific brain regions that are associated with a particular SNP.

**Figure 1:**
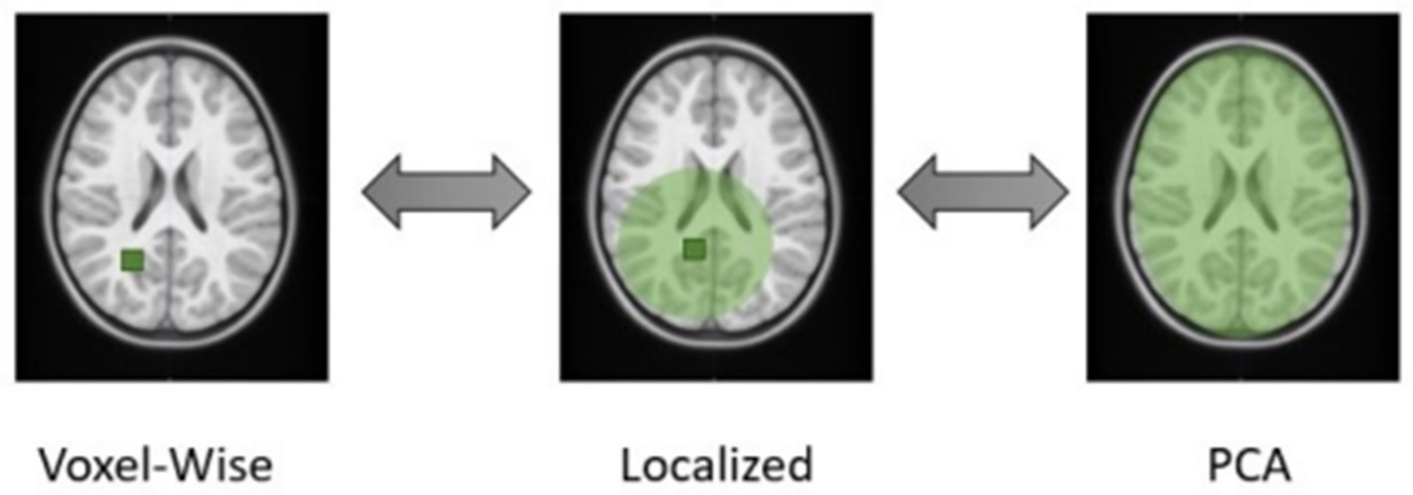
Schematic illustration of the different dimensionality reduction techniques. The proposed method closes the gap between voxel-wise and global PCA approaches, where the user defines a boundary distance, visualized by the green circle surrounding a particular voxel. This emphasizes how each component represents a different region of the brain.

We have recently proposed a more flexible localized approach (Dagasso et al., 2022) for integrating T1-weighted MRI datasets that falls on a spectrum between voxel-wise testing and global PCA (see Figure 1). The proposed approach performs a localized PCA on these MRI datasets via distance-based covariance matrix manipulations (Wilms et al., 2017). In doing so, components are more likely to encode more spatially localized information, which may be assumed to have higher associations with the genetic/genomic aspect of the analysis than a standard global PCA approach. This approach, therefore, effectively combines the strengths of a purely IDP-based dimensionality reduction for GWAS (ease of interpretation, use of prior knowledge) and PCA-based methods (fully data-driven). Another added benefit of the proposed PCA-like approach over voxel-wise testing schemes is its ability to generate data that visually highlights the morphological changes associated with the identified principal components. As in standard PCA, the localized principal components derived by our method span an affine subspace from which data can be sampled. With this generated data, a visual investigation of the viable morphometric brain traits is easily possible, which is useful from a clinical perspective to identify potential disease biomarkers. However, it remains to be investigated if the proposed method can generate meaningful results for mental health and neurodevelopmental disorders.

The aim of this paper was to extend the proposed method and further evaluate it in more detail within a first clinical feasibility analysis. Specifically, we aimed to investigate and provide evidence for the feasibility of our method and the strengths of the localized setup over a traditional PCA setup. Therefore, the proposed method was applied to data from the Adolescent Brain Cognitive Development (ABCD) (Casey et al., 2018) study to investigate if it can identify meaningful associations of specific morphometric brain features at varying levels of localization with genome regions when applied to data from adolescents with three different disorders with known genetic contribution. This manuscript presents a significant extension of Dagasso et al. (2022), with the major additions being: (1) implementation and evaluation of additional kernel sizes for the localized PCA, (2) an additional investigation of the proposed technique by application to three mental health and neurodevelopmental disorders, and (3) a greatly extended quantitative and qualitative evaluation of the results from a clinical perspective.

## 2 Materials and methods

### 2.1 Data

The 4th release of the Adolescent Brain Cognitive Development (ABCD) study data was used in this work to develop and evaluate the proposed localized morphometrics approach (Dagasso et al., 2022; Piras et al., 2015). The ABCD study is a longitudinal study conducted in the United States of America with initial enrolment of children between 9 and 10 years of age who are followed into early adulthood. ABCD contains imaging data, genomic data, and wide range of clinical and neuropsychological assessments. In this work, standardized T-scores from the Child Behavior Checklist (CBCL) DSM-5-Oriented Scale categories of attention deficit/hyperactivity problems, obsessive-compulsive problems, and depressive problems was used for definition of attention deficit hyperactivity disorder (ADHD), obsessive compulsive disorder (OCD), and depressive disorder, respectively. Therefore, for all three mental health and neurodevelopmental disorders, the CBCL T-scores were scaled to a range between 50 and 100, with a score of 50 representing the average for the subject's particular age and sex. Based on the CBCL scoring system, any scores falling below the 93rd percentile were considered normal for that category, scores falling in the 93rd to 97th percentile were in the borderline-clinical range, and scores above the 97th percentile were in the clinical range. For this work, borderline-clinical and clinical participants were grouped into the mental health or neurodevelopmental disorder class to maximize sample sizes, with the remaining participants falling under the 93rd percentile, for each of the CBCL DSM-5-Oriented Scale categories, comprising the non-disorders group (Stanley et al., 2022).

All available participants within the three identified categories of study in the disorder classes were included in this work provided existing quality-controlled genomic and medical imaging data were available. Individual age- and sex-based matching of controls to disorder groups were conducted for all three disorders, leading to variable sized control groups (see Table 1). Due to co-occurrence of diagnoses, it is possible that the same child can be assigned to more than one disorder group. Using the family ID's available, no individuals from the same respective family ID for each mental health and neurodevelopmental disorder setup were included more than once. To further maximize the sample sizes available, participants from minority groups in the ABCD study, specifically those self-identifying with non-white backgrounds were included. For each disorder dataset, ~80% of the participants were white (ADHD: 1,095, OCD: 1,357, depressive disorder: 1,367). The description of the number of participants included in each dataset is provided in Table 1.

**Table 1:** Participants included in each category for the neurodevelopmental disorder datasets.

	**ADHD (age range: 108–173 months)**			**OCD (age range: 107–171 months)**			**Depressive disorder (age range: 107–170 months)**		
	**Cases**		**Unaffected**	**Cases**		**Unaffected**	**Cases**		**Unaffected**
	**Subclinical**	**Clinical**		**Subclinical**	**Clinical**		**Subclinical**	**Clinical**	
Female	232	121	286	153	167	361	201	194	442
Male	220	135	365	274	246	551	166	212	551

#### 2.1.1 Genomics

The Affymetrix NIDA Smokescreen genotyping array (Ashburner et al., 1998) was used in the ABCD study as the sequencing platform for obtaining the genomic data, which contains 646,247 markers (SNPs) over 23 categories. Examples of the categories covered by this array include psychiatric disorders and common genes linked to addiction disorders. This array was specifically designed for investigations into smoking and other addiction behaviors with a particular focus on regions identified by various databases, such as HapMap, related to smoking behavior and nicotine metabolism and included 1,014 genes known to be associated with addiction (Ashburner et al., 1998). The quality-controlled genomic data includes samples from both saliva and whole blood to allow for higher successful calls and reduced missing data. Cleaning of the genomic SNP data was filtered for minor allele frequency (maf) of >0.05, with, the genotype missingness (geno) at 0.1, individual missingness (mind) at 0.1, exclusion of all variants with one or more multi-character allele codes (snps -only) and Hardy-Weinberg equilibrium (hwe) exact test *p*-being the default value set at 0.001 in PLINK (v2.0) (Smith, 2002). After filtering, 247,554 SNPs passed cleaning and filtering and were included in the subsequent genome-wide association studies described below.

#### 2.1.2 Magnetic resonance imaging

Among other sequences, structural T1-weighted MRI datasets with a resolution of 1.0 × 1.0 × 1.0 mm3 were acquired within the ABCD study using either Siemens, Phillips, or GE scanners at various field strengths. For further information about the specific details of the imaging acquisitions, we direct the reader to the detailed imaging protocol of the ABCD study (Casey et al., 2018). All imaging datasets used in this work underwent the ABCD minimal processing pipeline (Baurley et al., 2016), which follows common pre-processing standards such as bias field correction (Baurley et al., 2016).

### 2.2 Registration and deformation field generation

Instead of directly modeling the image content of the T1-weighted data, we restrict our analysis to morphological information using techniques from deformation-based, diffeomorphic morphometry to reduce the complexity of the analysis (Ashburner et al., 1998). The benefit of this approach is that it removes unwanted intensity differences between scans that may occur despite the harmonization of the imaging protocols between sites so that the analysis can be restricted to purely morphological differences. Therefore, we a brain extraction was performed in a first step to remove non-brain tissue from the images, using the Brain Extraction Tool (Smith, 2002). Next, each subject's T1-weighted image was registered to a common atlas, specifically the NIHPD asymmetric aged 7–11 years old (Fonov et al., 2011), using the non-linear, diffeomorphic image registration toolkit ANTs (Avants et al., 2008). The resulting deformation fields encode the morphological differences between the brain atlas and each subject's morphology on a voxel-by-voxel basis. Those deformation fields serve as a starting point for further analyses in this work. The final registrations for the participants were visually inspected to ensure proper registration of the patient data to the atlas template.

### 2.3 Localized PCA dimensionality reduction of the deformation fields

Principal component analysis (PCA) is a widely used multivariate technique for reducing the dimensionality of a dataset by identifying a low-dimensional affine subspace of maximum data variation. However, standard PCA does not account for the spatial relationships associated with medical imaging data. To address this limitation, we reduce the dimension of the vectorized deformation fields with our previously proposed spatially localized PCA approach (Wilms et al., 2017, 2022). Briefly described, instead of performing an eigen-decomposition of the sample covariance matrix, we manipulate the covariance matrix by reducing relations between field elements that are spatially far away in the image space. This helps us to focus on local information in the estimated PCA components. More specifically, the covariance matrix is manipulated with a distance-based Gaussian kernel function, whose width (distance parameter) can be selected by the user based on the Euclidean distance between image locations whose relationship should be preserved in the analysis. A more detailed description of this method can be found in Dagasso et al. (2022) and Wilms et al. (2017, 2022).

The distance parameters utilized in this work are defined in relation to the diagonal of the minimum-constraining bounding box surrounding the atlas being used for registration. The dataset used for the localized PCA was the participants' deformation fields from the registration step, with a training set defined as 80% of the total participants and 20% reserved for testing. The variability retained was set to 90% of the data for each of the chosen distance parameters, which defined the number of components used.

The generative modeling capabilities of a PCA-based morphometry model enable the exploration of the morphological data along various principal component axes, which can be used to our benefit in this context. More precisely, we can sample data from the estimated low-dimensional affine subspace, which enables a visualization of the morphological variation encoded by subspace directions/PCA components that are highly correlated to certain SNPs. Due to our unique and localized setup, the identified correlated components can be used to illustrate high structural variability within a specific, spatially localized region that might be otherwise lost in a global dimensionality reduction setup via standard PCA. Code availability at: https://github.com/wilmsm/localizedssm.

### 2.4 Multivariate genome-wide association study

Canonical component analysis (CCA) was performed for the multivariate GWAS in this work using mv-plink (Ferreira and Purcell, 2009). CCA aims to explain the largest possible amount of covariation between a SNP and all traits in the data by extracting a linear combination of all traits, and vice versa with the respective phenotypes. A multivariate setup was chosen in this work to allow for inherent correlations between the phenotypic features. Separate group comparisons were performed for each disorder described above and analyzed by using the localized components as endophenotypes to determine in which cases SNPs were more likely to be associated with morphological changes by comparing the participants in the unaffected groups and the participants in the disorder groups. For each component from the localized PCA setups described above, we adjusted this morphometric brain data for the covariates: sex, age, and the first 10 genetic principal components, which were calculated using PLINK v2.0 (Purcell et al., 2007). All principal components from the localized PCA setup for each disorder were included in these multivariate GWAS' in order to retain the whole brain representation from the localized PCA. The results of the multivariate GWAS' were visualized by Manhattan plots generated using the qqman R package (Turner, 2018).

### 2.5 Inversion of deformation fields and application to atlas

Following the analysis of the genome-wide association study results, components of the localized PCA that are more strongly correlated to each of the top SNPs were further investigated. The components most strongly correlated to a SNP in question were identified by setting an absolute value threshold, based upon the distribution of the results, of either >0.2 or 0.15, if there were none over 0.2. Exploration along the components axes within the affine subspace were then sampled to visually inspect the morphological variation encoded by this component.

### 2.6 Experimental setup

**Figure 2:**
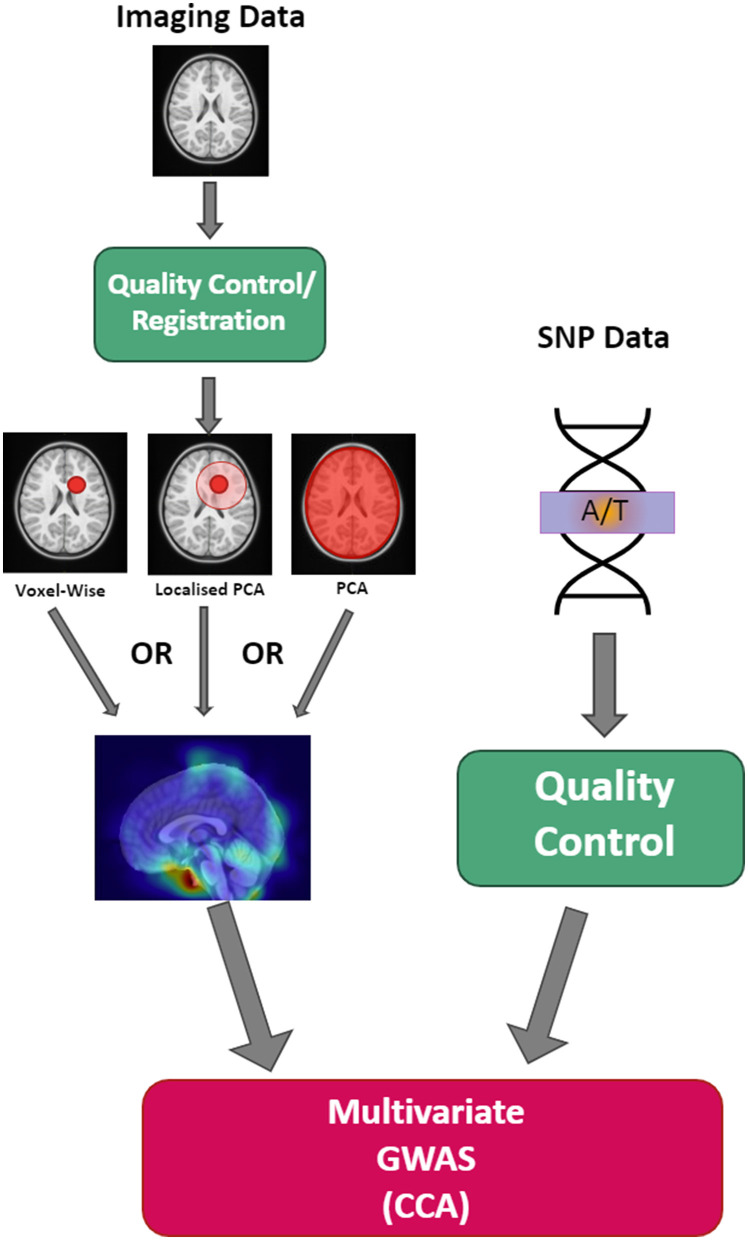
Overall schematic of the study setup.

Application of the methodology was done for three different mental health and neurodevelopmental disorders (ADHD, OCD, and depressive disorder) that are known to exhibit hereditary and morphological brain changes (Klein et al., 2019; Piras et al., 2015; Pauls, 2022; Zhang et al., 2018; Sayal et al., 2018; Wu et al., 2014; Hoogman, 2019). The distances chosen for each disorder were global, three-quarter, one-half, one-eighth, one-sixteenth, one-sixty fourth, and one-one twenty eighth distances. These distances were chosen to investigate a wide range of scales and to enable a comparison between localized PCA and global PCA. The one-one-hundred-twenty-eighth distance, in particular, represents a pseudo voxel-wise setup due to its fine resolution. A multivariate GWAS was computed individually for each of these distance parameters and for each mental health and neurodevelopmental disorder. The resulting components identified as being linked to genetic variants were then investigated to determine brain regions being stored within the component, which were identified using the CerebrA atlas (Manera et al., 2020). For the full methodology setup see Figure 2. Our study used a cluster node with 4x Intel(R) Xeon(R) Gold 6148 CPU and 3022 GB of RAM available with a runtime of ~6 h per distance threshold setup. For the multivariate GWAS setup, the same cluster node was used with a run time of ~24 h. We have added this information to our methodology section.

## 3 Results

### 3.1 Comparison of information stored within different distance setups

**Figure 3:**
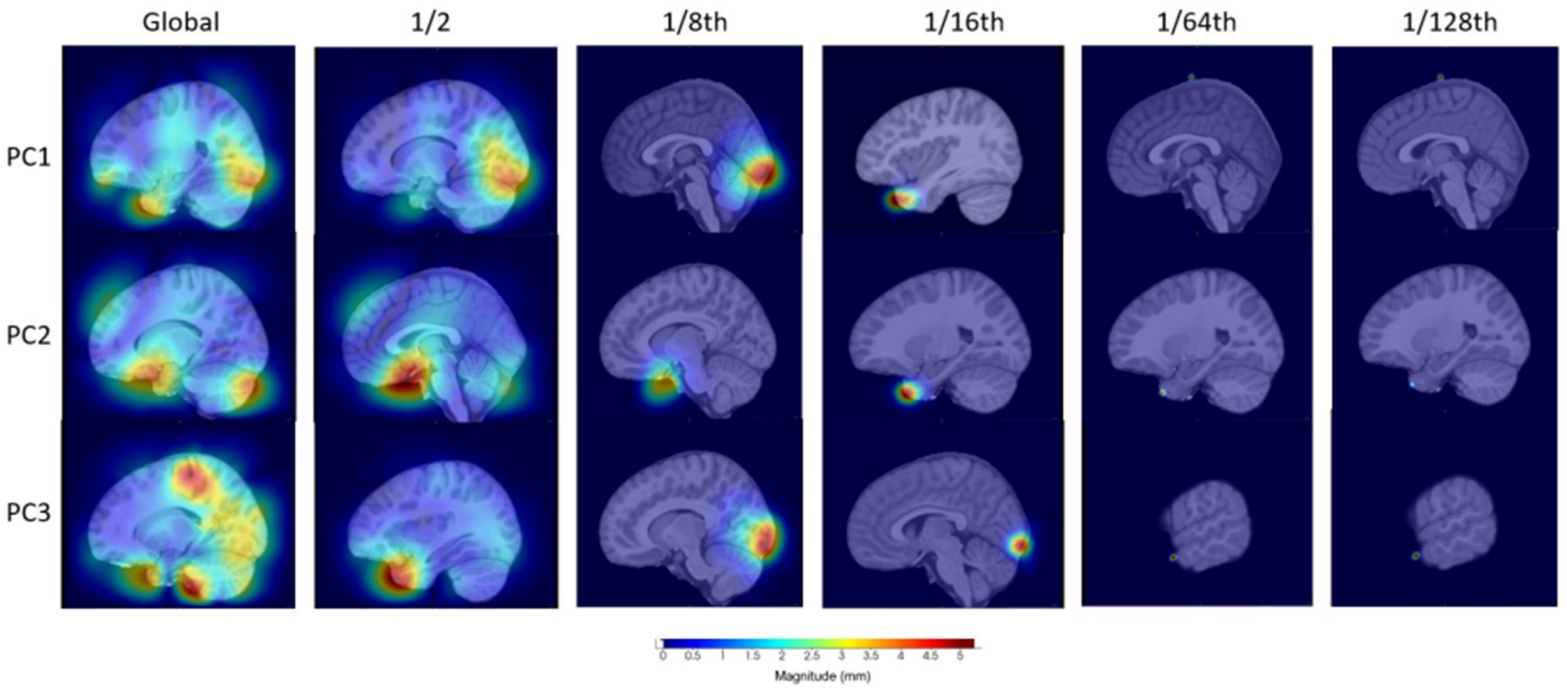
Visualization of the first three principal components for four distances to illustrate the differences in information stored for the OCD investigation.

To investigate the localization methodology, we visualized the information stored within the first three components for four of the distance parameter setups for children with OCD (see Figure 3). The sixteenth, eighth, half, and global distances were chosen in this example to illustrate the variability in the regions stored within the individual components as well as the localization of these magnitude changes in the deformation fields. As it can be seen, the global, or non-localized PCA, contains a large variability in regionality information stored in comparison with, for example, the one-sixteenth distance. This shows that the localized-based PCA-like setup can represent and analyze more regional based information. Moreover, it is worth noting that when using smaller distance parameters, such as 1/64th or 1/128th, it is becoming increasingly difficult to identify specific regions of importance and as such may be similar to voxel-wise testing with its problems in this sense.

Moreover, Table 2 provides the number of components that were retained for each neurodevelopmental disorder for each distance setup. In comparison to the ABCD tabulated structural imaging data, which contains over 450 radiomic features, such as cortical surface area and thickness measurements from the Destrieux atlas (Fischl, 2012), this illustrates the dimensionality reduction capabilities of our setup.

**Table 2:** Number of components used per analyses for each disorder included in our experimental setup.

**Number of components per distance set-up**	**ADHD**	**OCD**	**Depressive disorder**
Global	265	226	225
3/4	249	234	233
$\frac{1}{2}$	268	249	249
1/8	348	343	345
1/16	320	326	324
1/64	282	283	285
1/128	268	279	280

### 3.2 Attention-deficit hyperactivity disorder

**Figure 4:**
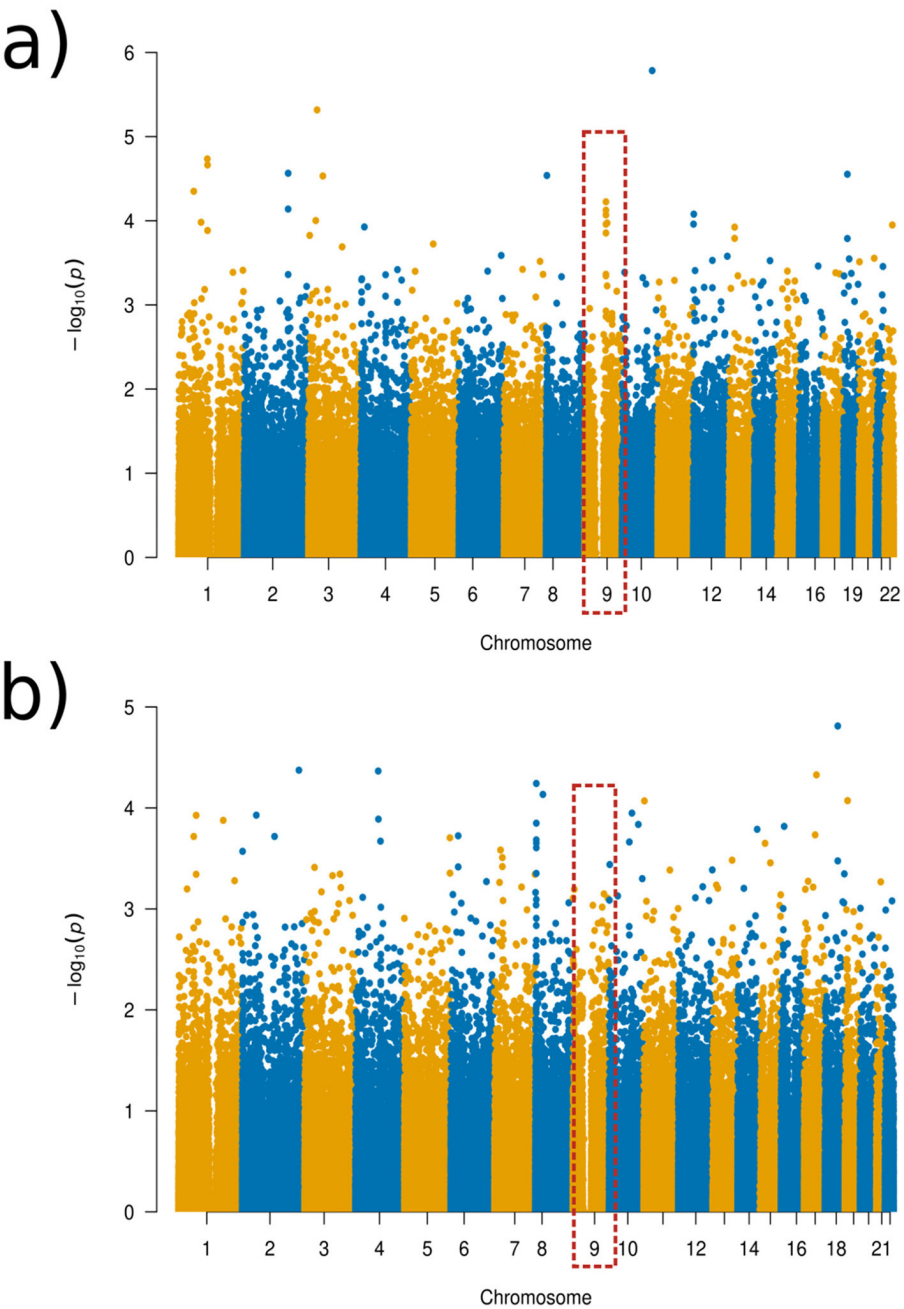
Manhattan plots of the GWAS results for the ADHD experimental set-up. **(a)** Global distance set-up Manhattan plot. **(b)** 1/64 distance set-up Manhattan plot.

For the experiment with the ADHD group, we found that the more localized distance setups result in additional genotype-phenotype associations that were not identified in the global distance setup that practically equals a standard PCA. This is illustrated in Figure 4 while additional visualizations are available in Supplementary Figures 1–7.

**Figure 5:**
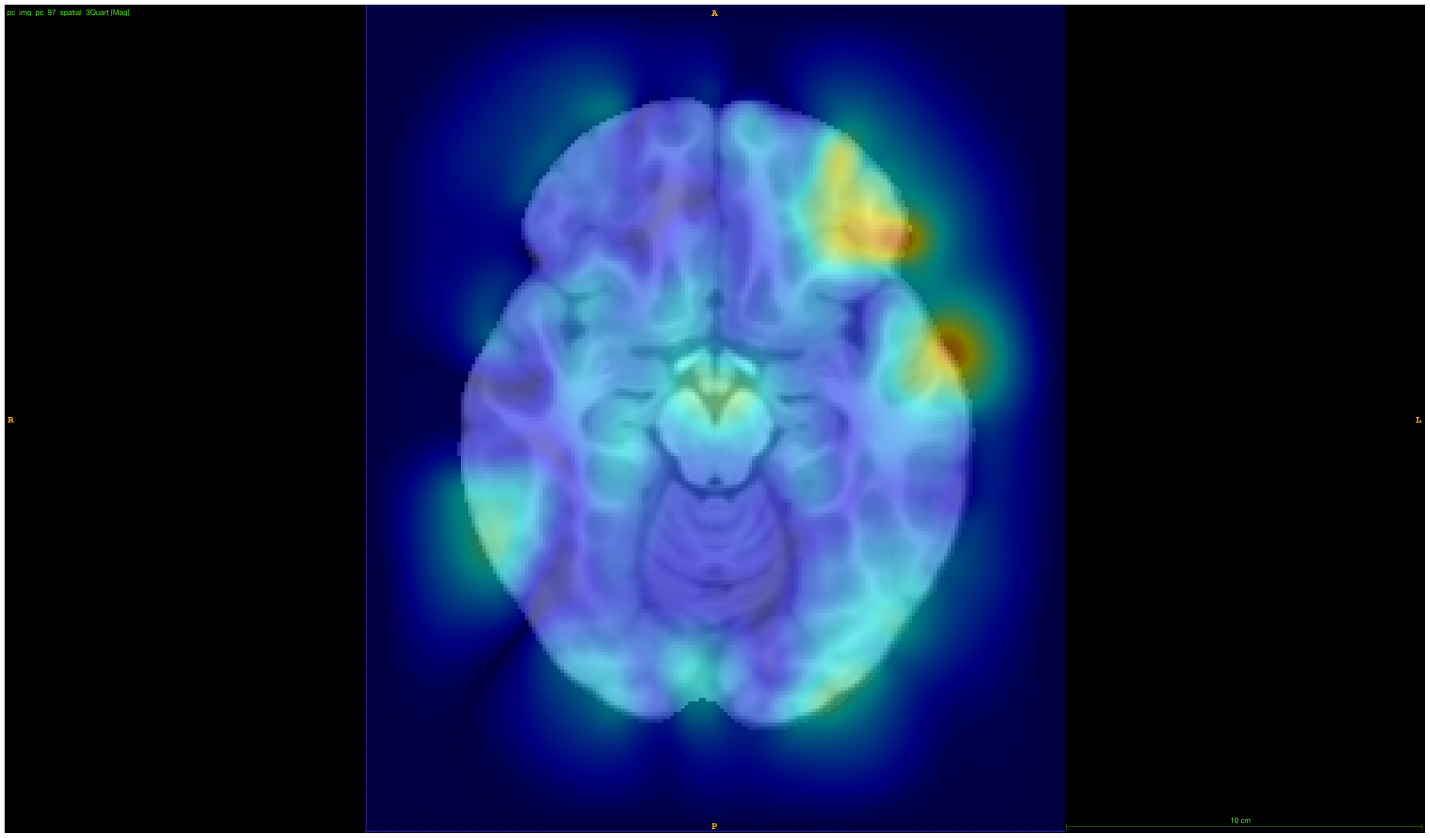
Visualization of the axial view for component 97, in the 3/4 distance set-up.

Using Manhattan plots, we identified two genomic regions on chromosome 8 and on chromosome 9 that were further investigated. Although these regions did not reach genome-wide significance, we identified these SNPs as being the most significant in the Manhattan plots towers in the following and, therefore, as potential targets for future studies. More precisely, for the global distance and the other larger distance setups, we investigated a region on chromosome 9 around the gene GNAQ, for the three-quarter distance, we particularly investigated the SNP rs1930541 (*p*-value 1.24 × 10^−5^), which is an intron variant in the gene GNAQ, which has ubiquitous gene expression levels. However, this gene or SNP has not been priorly implicated in ADHD or neurodevelopmental disorders, which makes it an interesting target for future studies. The 97th component was strongly linked to this SNP, and the left hemisphere pars orbitalis and the middle temporal regions had the highest changes of magnitude within this component (see Figure 5). Volume changes in the pars orbitalis and middle temporal regions have been previously (Nickel et al., 2018; Shaw et al., 2007).

**Figure 6:**
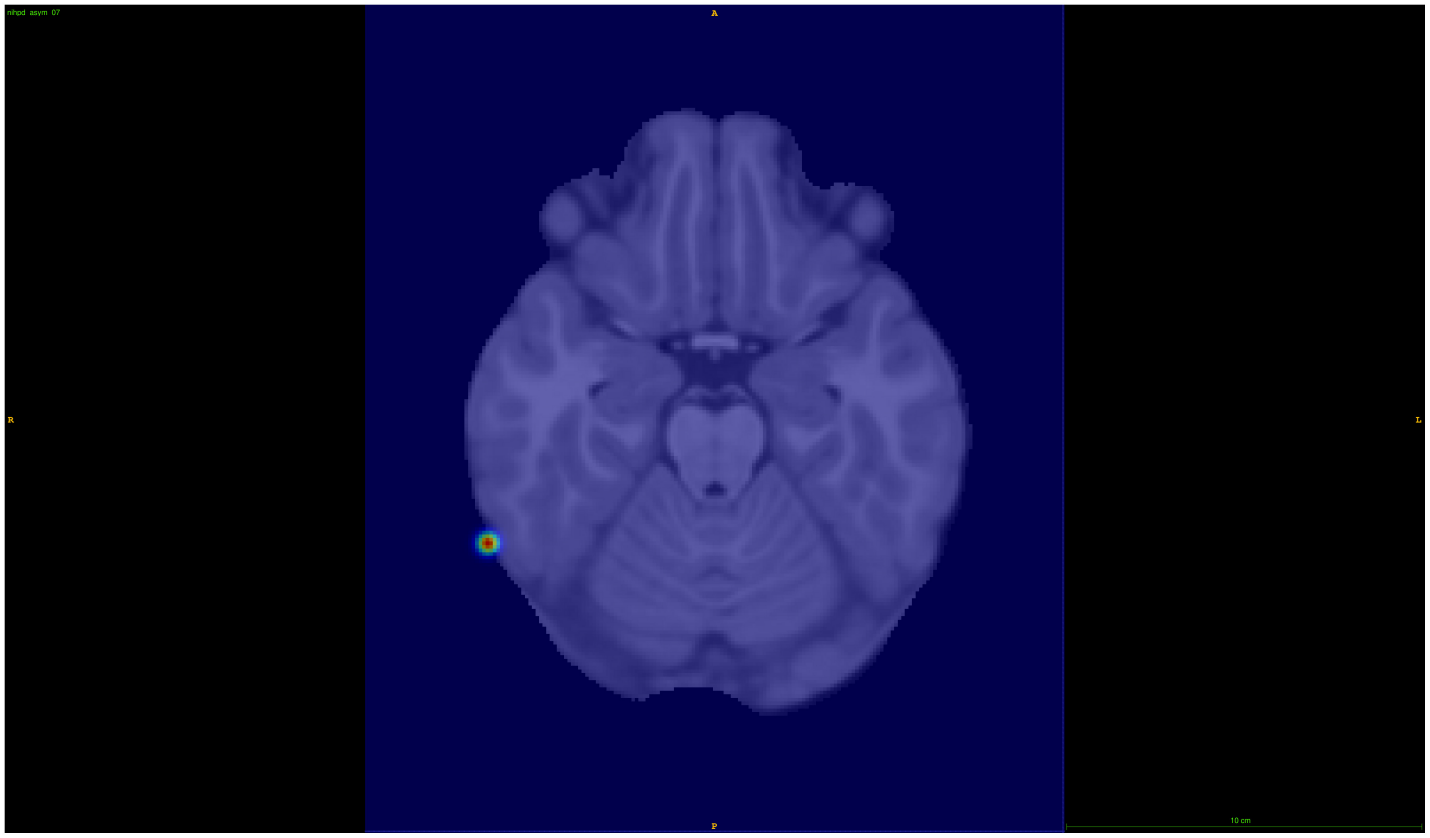
Visualization of the axial view for component 68, in the 1/64 distance set-up.

For chromosome 8, we investigated the results from the 1/64 distance setup, with the SNP rs6998882 (*p*-value 5.72 × 10^−5^) being the most significant in this region. This SNP is an intron variant in the gene region of CSMD1, which has been previously reported in ADHD-related studies (Liu et al., 2021), and has a biased brain expression. The 68th component was further investigated in this case, showing to be associated with the right hemisphere's inferior temporal brain region (see Figure 6).

### 3.3 Obsessive-compulsive disorder

**Figure 7:**
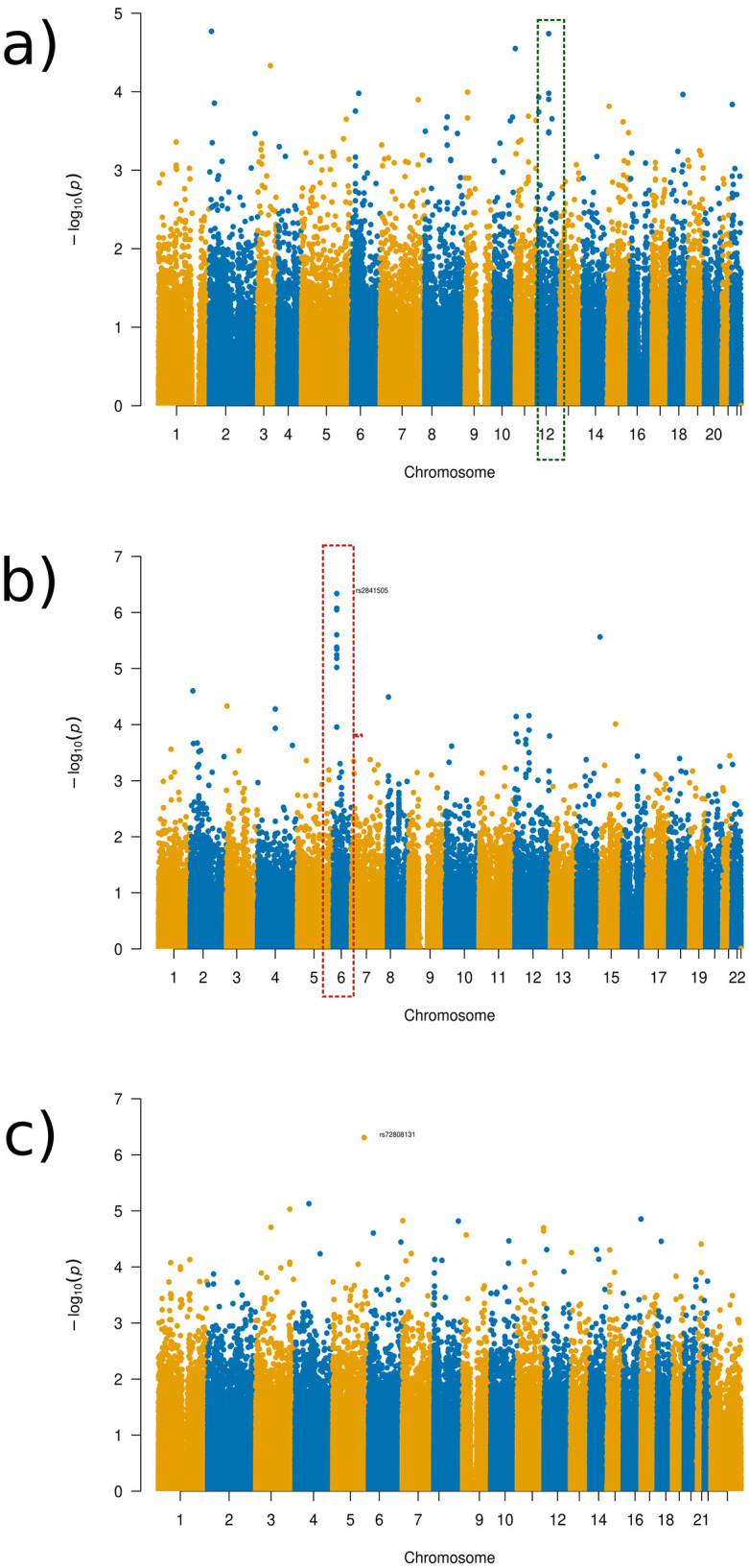
Manhattan plots of the GWAS results for the OCD experimental set-up. **(a)** Global distance set-up Manhattan plot. **(b)** 1/16 distance set-up Manhattan plot. **(c)** 1/128 distance set-up Manhattan plot.

Visualizations from the Manhattan plots illustrate how different distance parameters provide varying information or more clear patterns depending on the distances used for the patients with obsessive-compulsive disorder (see Figure 7). In the 1/8 distance setup, for example, there is a clear tower, which was not visible as clearly in the global distance, though with the finer distance setups, these clear patterns seem to disappear (see Figure 7; Supplementary Figures 8–14).

As can be seen in the Manhattan plots, no SNPs found were at a genome-wide significance level (1 × 10^−8^). Despite this finding, we discuss some SNPs that were identified as being the most significant in the Manhattan plots towers in the following as potential targets for future studies. The SNPs were chosen for discussion as the towers indicate that a more likely associated SNP occurs in this region that is more likely associated despite not being significantly linked to the brain regions in question. The tower seen in chromosome 6 in varying distance setup Manhattan plots occurred in the gene SIRT5, with SNP rs2841505 (*p*-value 4.75 × 10^−6^) being the most significant SNP in that region, as visualized in the Manhattan plot for the 1/16 distance (see Figure 7b, dotted red rectangle). SIRT5 has an increased level of brain tissue expression levels (Carithers et al., 2015) but has not been priorly implicated in OCD or other neurodevelopmental disorders so far. For the global distance setup, we investigated chromosome 12, which occurs within the LRRK2 gene, as highlighted by the green rectangle in Figure 7a. This result is also apparent within the half-distance setup, although with more noise mixed in within the top SNPs. LRRK2 has been priorly implicated in Parkinson's disease and has been noted to have an effect on dopamine receptor trafficking (Rassu et al., 2017). In line with this finding, current research suggests a link between OCD and dopamine pathways (Dong et al., 2020). LRRK2 was found to have an ubiquitous tissue expression (Carithers et al., 2015).

**Figure 8:**
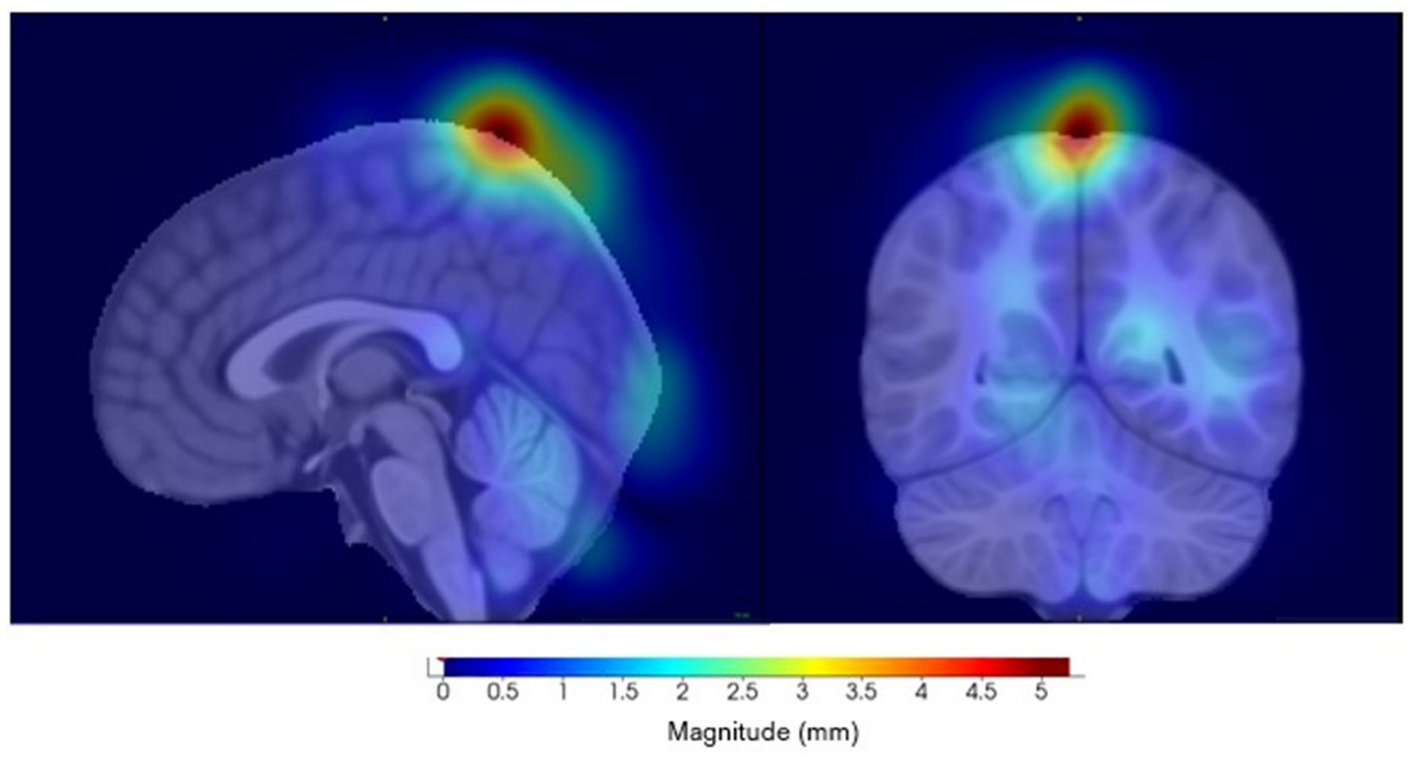
Visualization of component 13, for the eighth distance set-up, highly correlated to SNP rs11564150. **(Left)** Sagittal view. **(Right)** Coronal view.

The component with the highest link to the top SNP, rs11564150 (*p*-value 1.82 × 10^−5^), in LRRK2 was component 13 (see Figure 8). This component was mainly associated with the precuneus region in both, the right and left hemisphere, showing major deformation changes. The precuneus region has been previously linked to OCD (Piras et al., 2015). All distance setup Manhattan plots not shown in the main paper can be found in Supplementary material.

### 3.4 Depression

**Figure 9:**
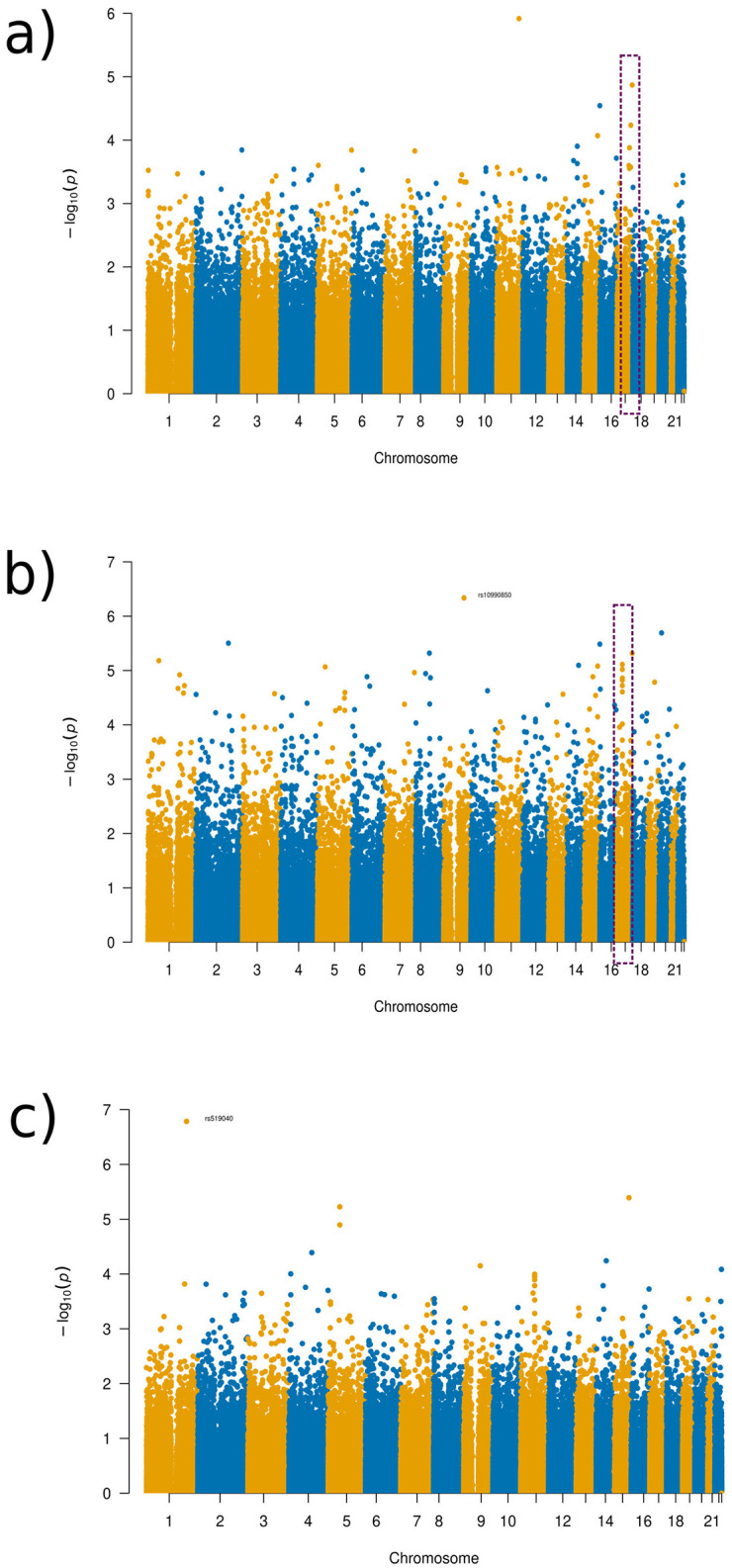
Manhattan plots of the GWAS results for the depression disorder experimental set-up. **(a)** Global distance set-up Manhattan plot. **(b)** 1/8 distance set-up Manhattan plot. **(c)** 1/128 distance set-up Manhattan plot.

Similar results as in OCD for comparing SNP results across the different distances were found in the depression group. As can be seen in the Manhattan plots for the varying distances (see Figure 9; Supplementary Figures 15–21), there were varying results across the distances. However, a relatively consistent region in chromosome 17 can be identified in several of the plots (see Figure 9, purple rectangle), with a clear distinctive tower, which was the reason for further investigation, despite not meeting genome-wide significance. All distance setup Manhattan plots not shown in the main paper can be found in Supplementary material.

The region in chromosome 17 identified occurred in the gene AATK, with the SNP rs2725417 (*p*-value 4.82 × 10^−6^) from the 1/8 distance being the most significant SNP in this region. This gene has biased brain expression but has not been priorly implicated in any mental health or neurodevelopmental disorders so far.

**Figure 10:**
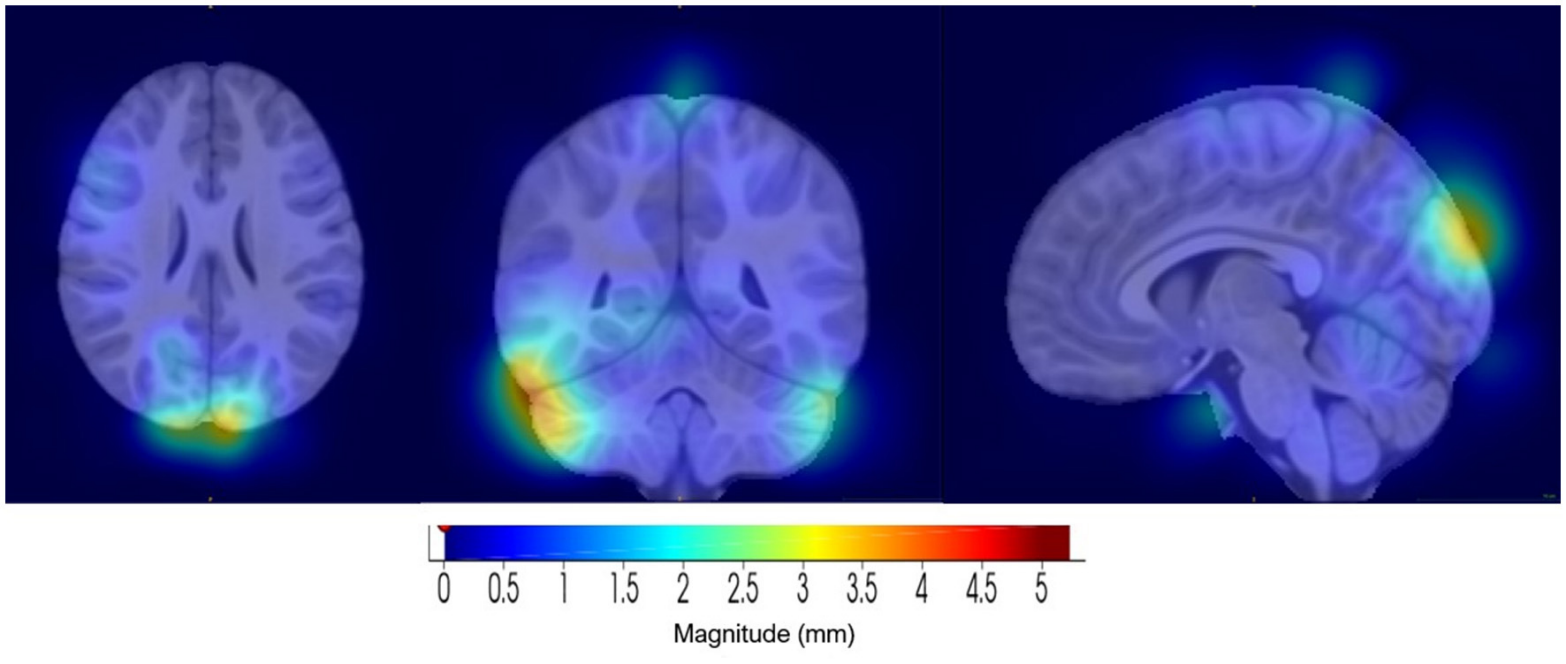
Visualization of component 20, for the 1/8 distance set-up, which was highly correlated with SNP rs2725417. **(Left)** Axial view. **(Middle)** Coronal view. **(Right)** Sagittal view.

Component 20 (see Figure 10) showed the highest link to SNP rs2725417 and includes part of the cerebellum, inferior temporal, and the left and right lateral occipital regions. Alterations in the cerebellum region, including structural and functional, have been implicated in several psychiatric and neurodevelopmental disorders, including depression. However, the exact mechanisms how the cerebellum is affected remain unclear in depression (Phillips et al., 2015; Depping et al., 2018; Sathyanesan et al., 2019; Moberget et al., 2019).

## 4 Discussion

Imaging genetics is a relatively new field that is still exploring how to best include and combine genotype and phenotype datasets in a unified analysis, while ensuring both (1) the proper use of the datasets and (2) the full realization of the potential both data modalities have for current and future studies. Given the sheer size of both datasets, with millions of voxels in MRI datasets, and potentially hundreds of thousands to millions of SNPs available, the development of new approaches for targeted, effective image dimensionality reduction for GWAS' is an important avenue of research. In this study, we investigated the utility of a novel and efficient method to include whole brain image information in a GWAS and further tested our method on three different pediatric mental health and neurodevelopmental disorders. The proposed method combines ideas from deformation-based morphometry and localized PCA. We showcase that the localized dimensionality reduction approach provides an opportunity to investigate specific, spatially-localized, imaging-derived phenotypes that can be used in conjunction with existing multivariate genome-wide association study frameworks in a data-driven way without having to rely on pre-defined atlas-based parcellation of the brain space. This is especially beneficial in cases where medical and mental health conditions or disorders may affect more than one region of the brain in different (non-linear) ways.

The approach presented in this work can be broadly applied to various disorders to generate new hypotheses that can then be tested in more detail later on by using various distance thresholds. By doing this, no assumptions are made what features are linked to various medical and mental health diseases or disorders, which separates it from other setups that utilize atlases or masking of particular brain regions. In addition, the ability to work with a range of distances, this method can help identify further genotype-phenotype associations that would otherwise be undetectable as shown in this work for three specific disorders. Along the same lines, the proposed method is flexible enough to encode additional prior knowledge about brain morphology into the pipeline (e.g., hemispheric symmetries) by adjusting the distance measure used accordingly.

The generative modeling aspect of this pipeline is an added benefit, given its ability to visualize phenotypic changes associated with correlated components along their axes. This visualization may be useful when analyzing the morphological changes that are highly associated with a SNP. This could be potentially useful for future precision medicine applications investigating how a particular SNP causes morphometric effects on a patient level. Moreover, it allows to visualize specific changes that one is unable to display when using data such as Jacobian matrices (Rodrigue et al., 2020) or even voxel-wise testing (Stein et al., 2010), the two other techniques often used in GWAS analyses of neuroimaging data.

We demonstrated the utility of this pipeline in directly associating morphometric, imaging-derived phenotypes from T1-weighted imaging with genomic regions in preadolescent participants with and without ADHD, OCD, and depressive disorder, all of which are hereditary disorders with some known or previously reported structural brain changes. It should be emphasized that our setup, while applied to T1-weighted imaging datasets in this work, can be theoretically applied to any other brain imaging modality or sequence as well and can be combined with other statistical tests. Examples of other acquisition techniques could be T2-weighted imaging, diffusion tensor imaging to obtain connectivity information, which could be especially important in disorders such as ADHD like in our study, or functional magnetic resonance imaging. The overall motivation of this study was to provide a more intuitive way to include diverse high dimensional imaging data in GWAS', as such our method is not limited to macro-structural brain imaging data and can also be employed in multi-modal and other non-brain imaging contexts (e.g., cardiac imaging).

The findings from the various distance parameter ADHD configurations revealed a strong correlation in the middle temporal and pars orbitalis regions, brain regions that have been previously associated with ADHD (Nickel et al., 2018; Shaw et al., 2007). Likewise for the OCD cohort, the proposed method led to feasible results with a relevant SNP identified in the gene region LRRK2, which is implicated in dopamine receptor trafficking, which is a feasible result given recent research (Dong et al., 2020). For the third disorder cohort, depressive disorder, we also found a feasible result with the cerebellar region having been reported to be implicated in psychiatric as well as neurodevelopmental disorders.

Overall, the SNP results from the GWAS' found more distinct patterns within the 1/16 and 1/8 distance setups in comparison to smaller distance parameter setups. This may indicate that as the distance parameters are reduced, we are reaching a lower limit of distance parameters that is needed or leads to reasonable results. Thus, using very small distance setups that are more similar to voxel-wise analyses may not reveal any additional insights and may even hide them. Given the variability of the top SNPs in the larger distances, global or 1/2 for example, compared to 1/8, there may be SNPs that are more highly linked to the global changes across the brain structures in comparison to SNPs that are more local to specific brain structure changes, again directly showing a benefit of the proposed method. Thus, using a top-down approach using distances such as global, 1/2, and 1/16 may be useful to investigate more global structural changes to more specific structural changes (this was visualized in Figure 3).

One of the main limitations of this study are the relatively small sample sizes for each of the included neurodevelopmental and mental disorders, which is the reason why we chose to enhance our sample sizes by including subclinical groups. This limitation is well-recognized in pediatric datasets, where obtaining large, comprehensive datasets can be challenging. In the future, we aim to address this issue by scaling our work to adult datasets, as larger and more robust datasets are typically available for adult cohorts. This will allow us to investigate the generalizability of our findings and validate them in a larger dataset. Additionally, given the heterogeneity of study sites, there may be site-specific biases contained in the imaging. However, the ABCD study did harmonize and optimize imaging acquisitions across the three scanner platforms (Casey et al., 2018). While other global PCA-like methods (Mihalik et al., 2022; Anderson et al., 2014; Arnedo et al., 2015), which focus on global eigenvalue decomposition of the data and other atlas-based analyses exist, a comparison between our localized dimensionality reduction technique and these methods is currently out of scope for this study as the focus of our paper was to present and provide a first feasibility analysis of our method to identify the potential benefits of a localized setup.

The localized PCA setup described in our study enables the inclusion of full images via morphometric, imaging-derived phenotypes from T1-weighted imaging into multivariate GWAS frameworks. The results found were overall clinically feasible and in line with current knowledge in the domain, but may also indicate new findings for the neurodevelopmental disorders included in this work. Overall, our method holds considerable promise for investigations of data-driven imaging phenotypes in a multivariate GWAS setup for identifying new genotype-phenotype associations, which can be applied to other diseases and disorders.

## 5 Conclusion

This proposed approach is a novel fully-data driven methodology that enables the inclusion of any medical imaging data without the need for pre-definition of spatial regions into a multivariate GWAS. The findings for the three psychiatric and neurodevelopmental disorders we tested it on are feasible in terms of the genotype and phenotype characteristics, both. While we showcase its capabilities on neuroimaging data, our method and its associated pipeline can be applied to any type of medical imaging data to support manifold genotype-phenotype analyses that may help to identify unknown genomic variants. The minimum number of images needed for this analysis depends on the signal in the data and cannot be generally defined by a single number.

## Data Availability

The original contributions presented in the study are included in the article/Supplementary material, further inquiries can be directed to the corresponding author.
