# Supplementary material for: Application of a localized morphometrics approach to imaging-derived brain phenotypes for genotype-phenotype associations in pediatric mental health and neurodevelopmental disorders
Source: Front Big Data. 2024 Dec 11;7:1429910. doi: 10.3389/fdata.2024.1429910 (PMC11668761; doi:10.3389/fdata.2024.1429910)
Supplement: Supplementary file 1 [file Data_Sheet_1.pdf]

Figure 11. Manhattan plot for the eighth distance set-up for the obsessive compulsive disorder dataset.

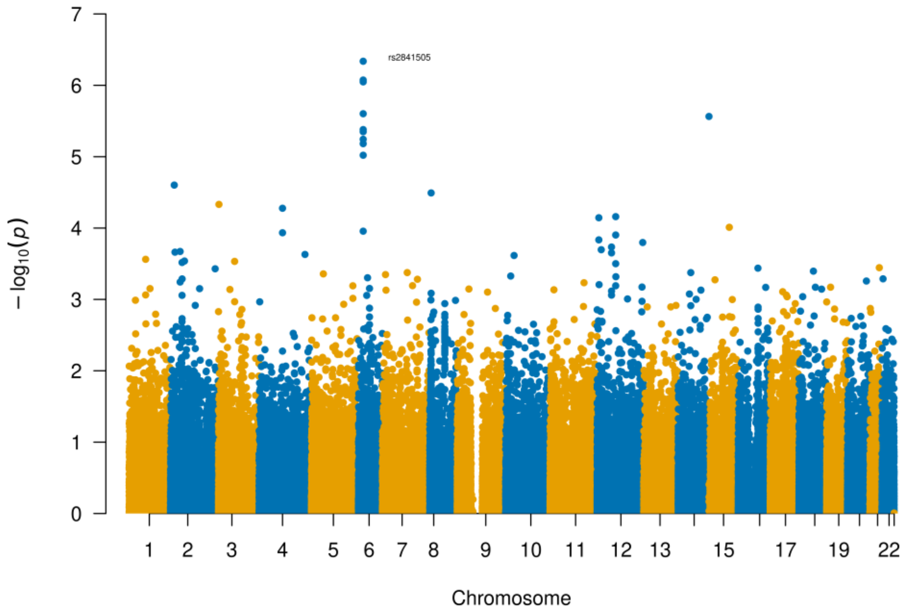

Figure 12. Manhattan plot for the one-sixteenth distance set-up for the obsessive compulsive disorder dataset.

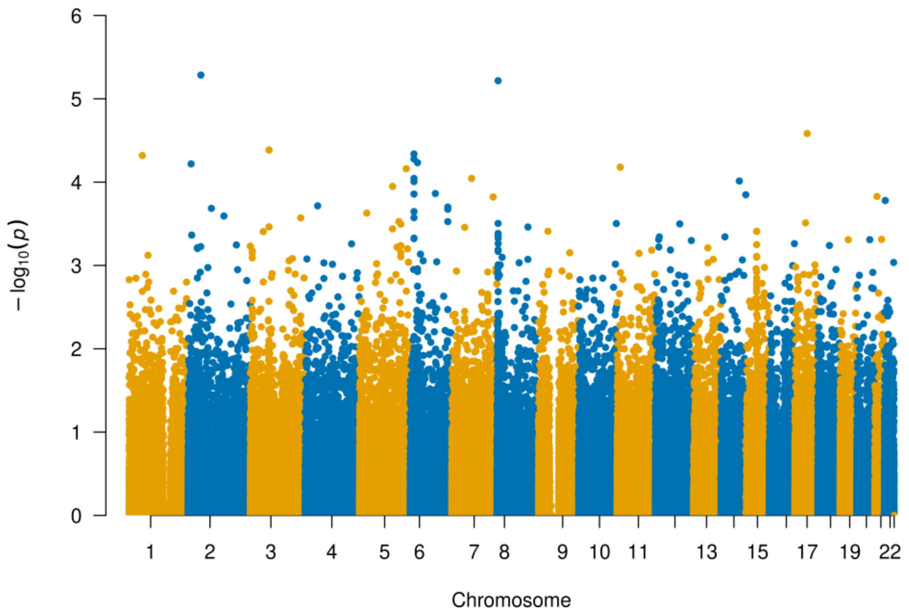

Figure 13. Manhattan plot for the one-sixty fourth distance set-up for the obsessive compulsive disorder dataset.

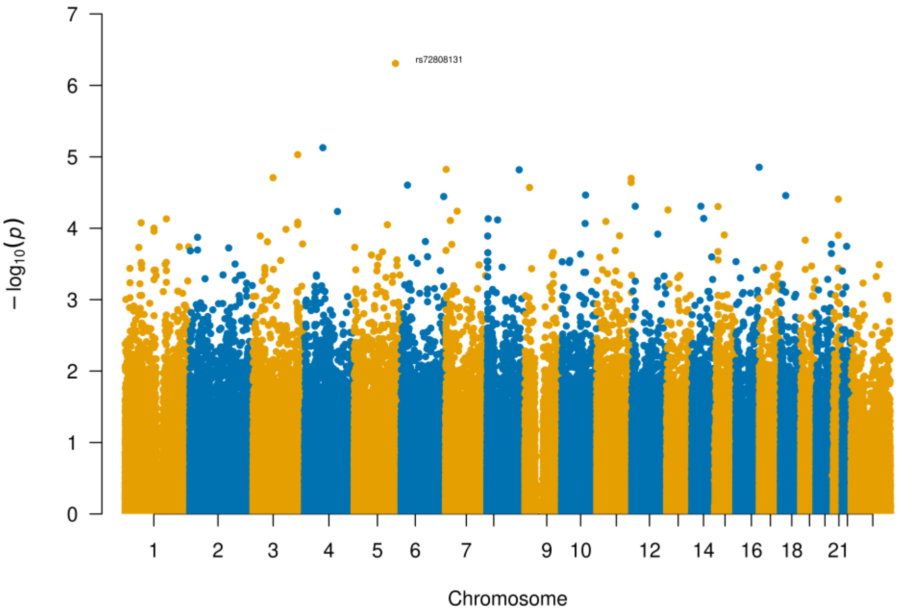

Figure 14. Manhattan plot for the one-one twenty eighth distance set-up for the obsessive compulsive disorder dataset.

Depressive Disorder Manhattan Plots

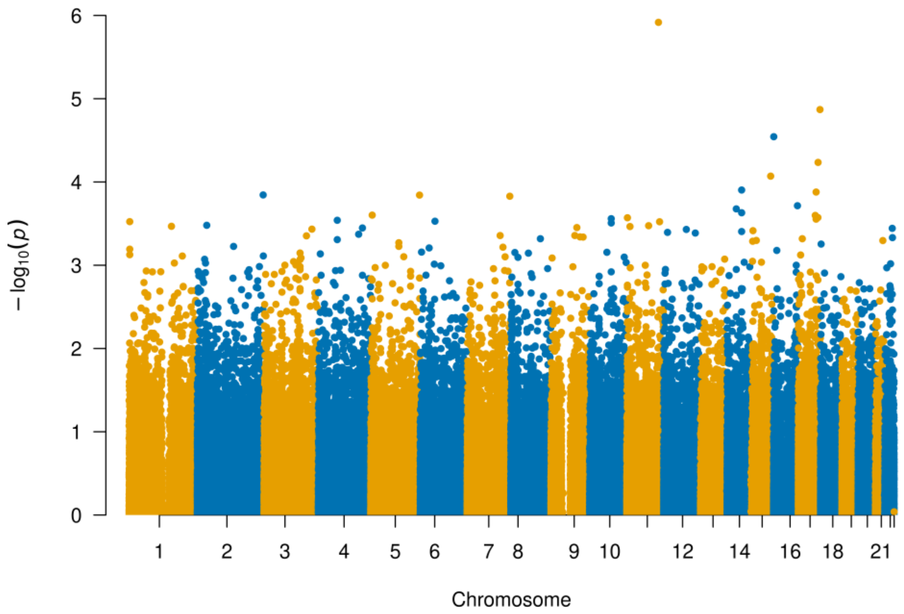

Figure 15. Manhattan plot for the global distance set-up for the depressive disorder dataset.

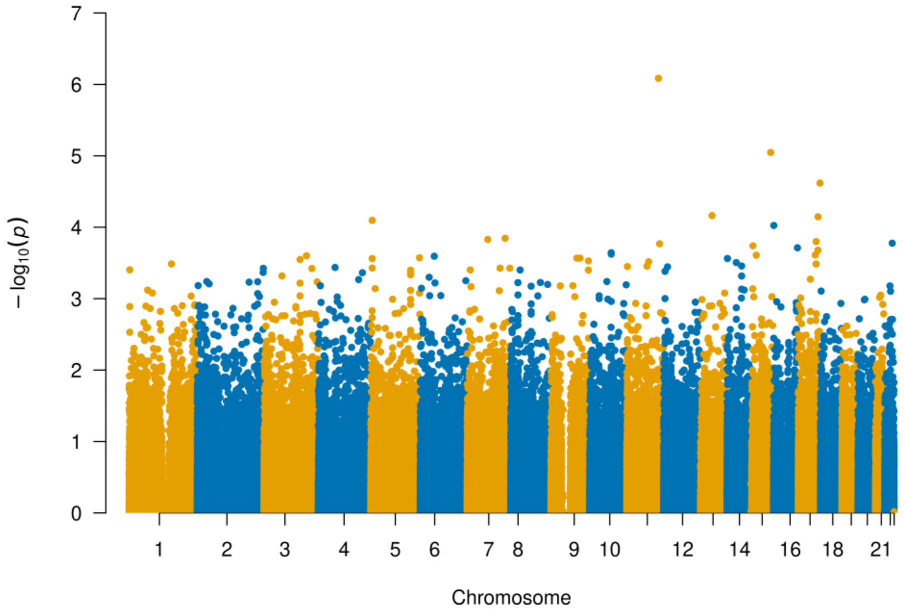

Figure 16. Manhattan plot for the three-quarter distance set-up for the depressive disorder dataset.

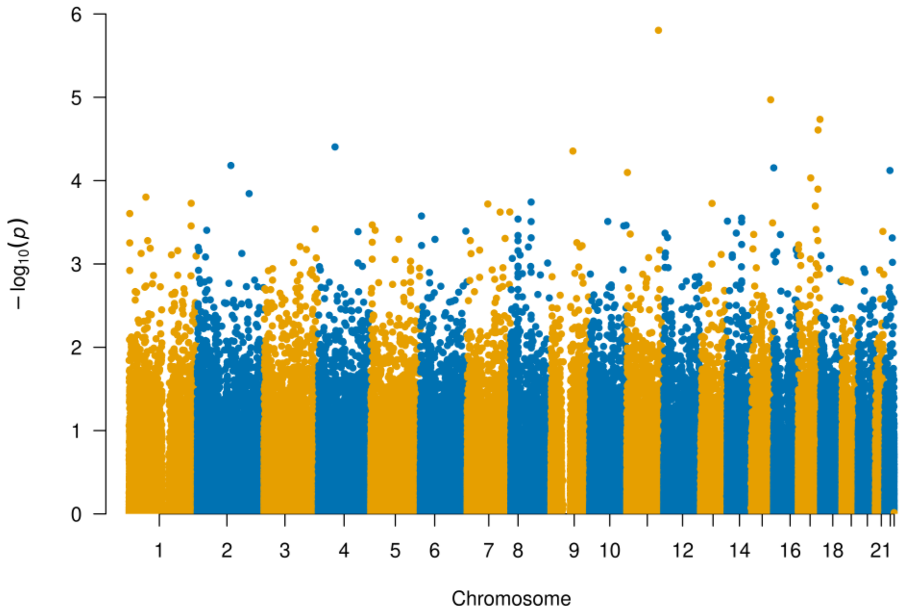

Figure 17. Manhattan plot for the half distance set-up for the depressive disorder dataset.

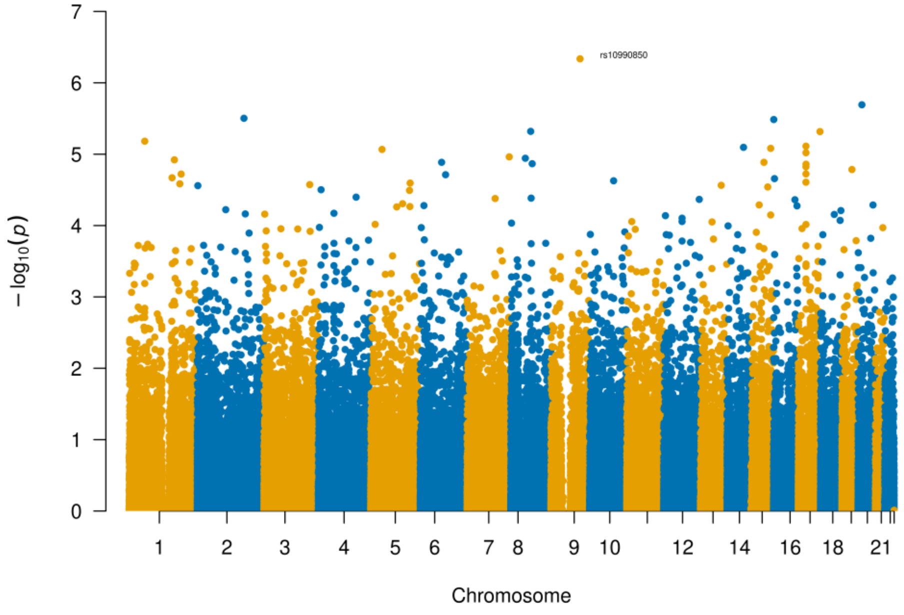

Figure 18. Manhattan plot for the eighth distance set-up for the depressive disorder dataset.

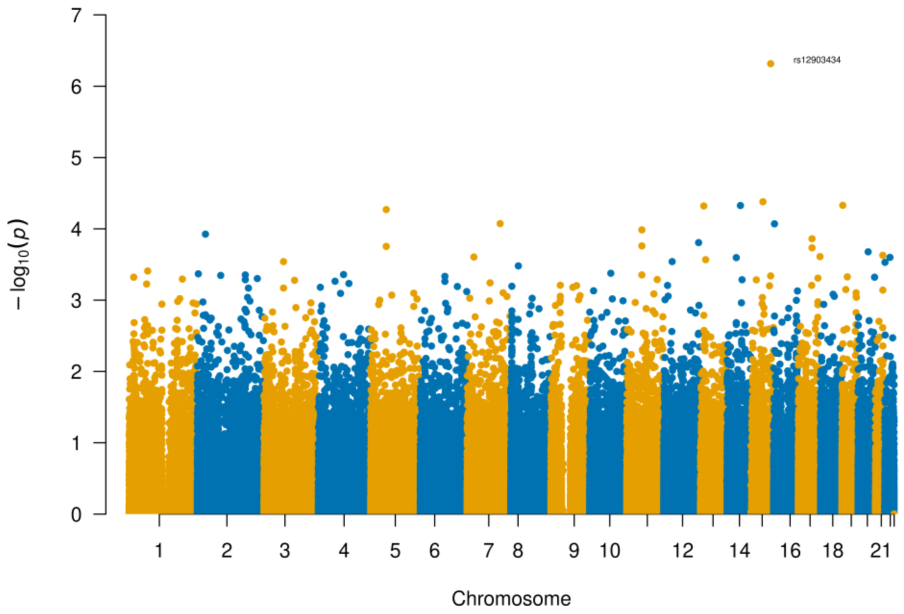

Figure 19. Manhattan plot for the one-sixteenth distance set-up for the depressive disorder dataset.

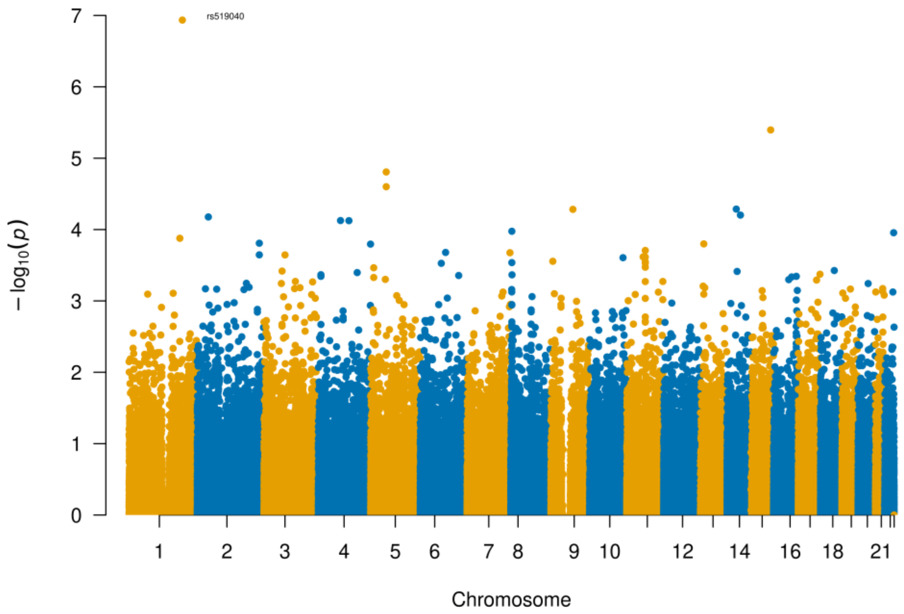

Figure 20. Manhattan plot for the one-sixty fourth distance set-up for the depressive disorder dataset.

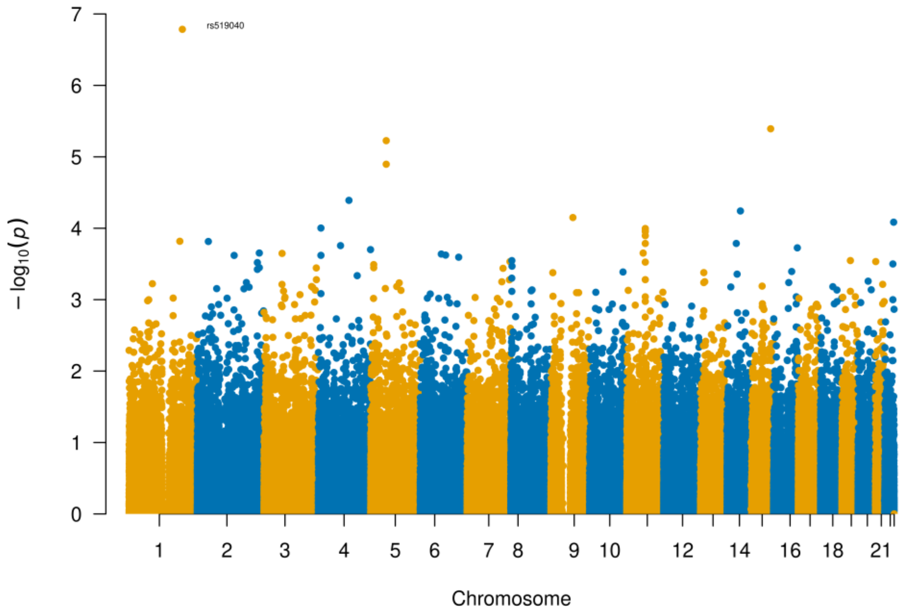

Figure 21. Manhattan plot for the one-one twenty eighth distance set-up for the depressive disorder dataset.
