# Supplementary material for: Application of a localized morphometrics approach to imaging-derived brain phenotypes for genotype-phenotype associations in pediatric mental health and neurodevelopmental disorders
Source: Front Big Data. 2024 Dec 11;7:1429910. doi: 10.3389/fdata.2024.1429910 (PMC11668761; doi:10.3389/fdata.2024.1429910)

Attention-Deficit Hyperactivity Disorder Manhattan Plots

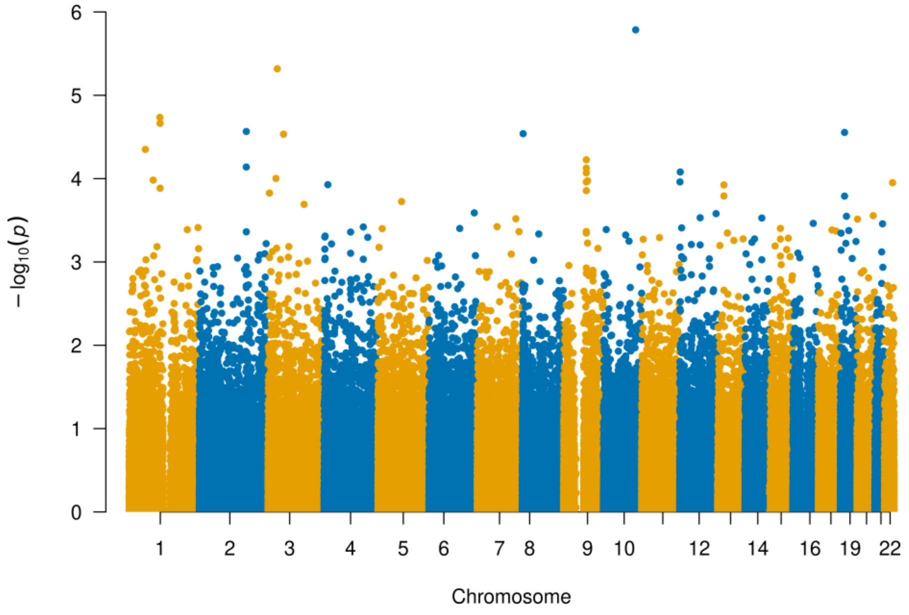

Figure 1. Manhattan plot for the global distance set-up for the attention-deficit hyperactivity disorder dataset.

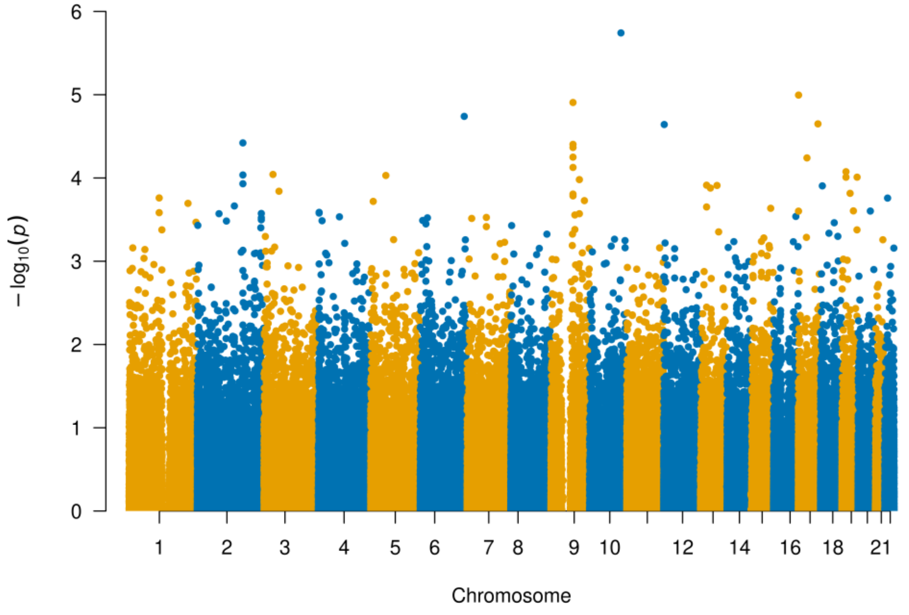

Figure 2. Manhattan plot for the three-quarter distance set-up for the attention-deficit hyperactivity disorder dataset.

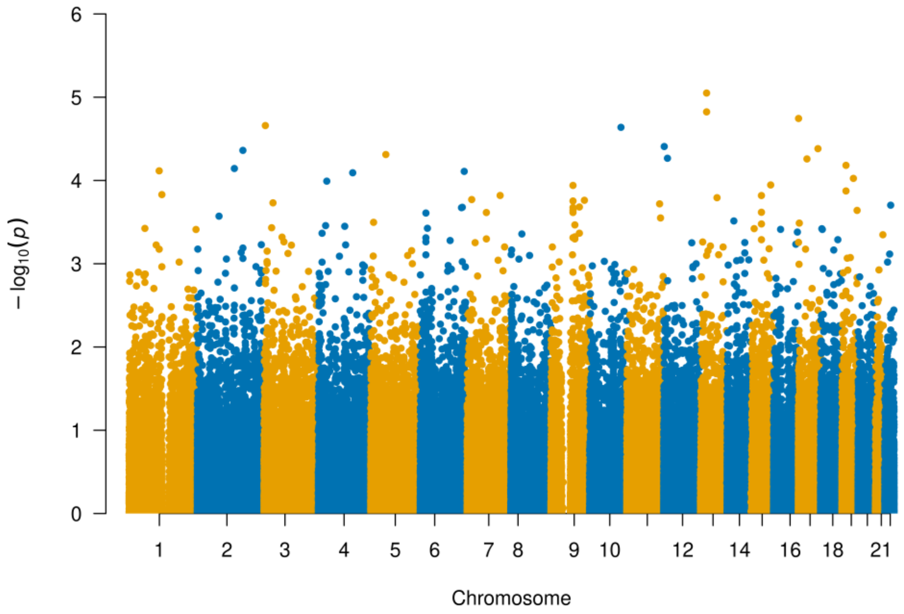

Figure 3. Manhattan plot for the half distance set-up for the attention-deficit hyperactivity disorder dataset.

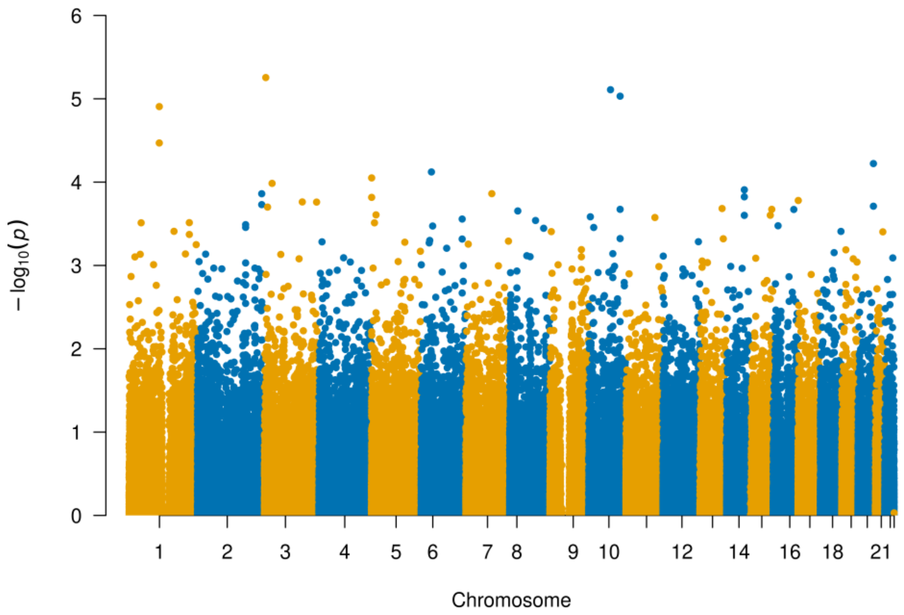

Figure 4. Manhattan plot for the one-eighth distance set-up for the attention-deficit hyperactivity disorder dataset.

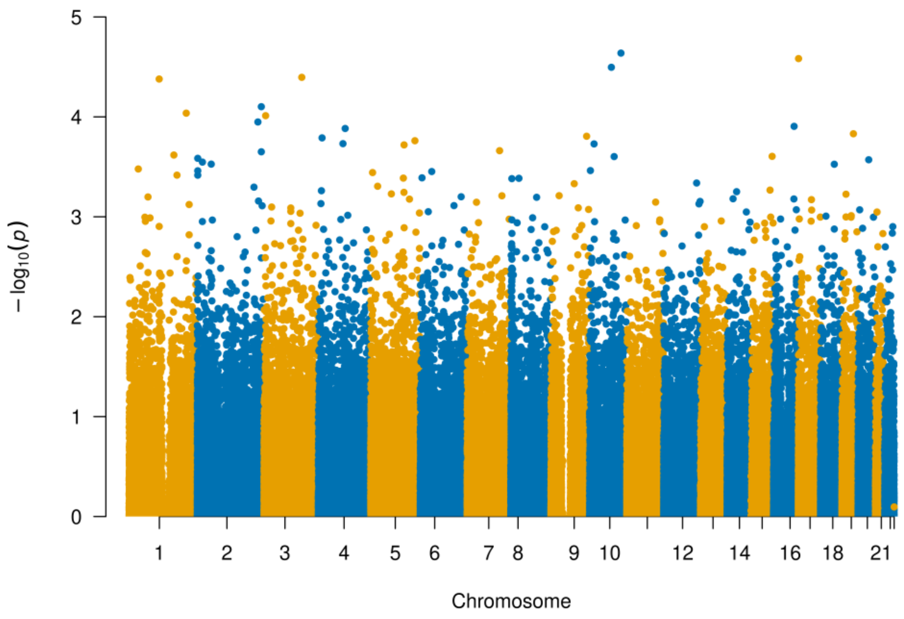

Figure 5. Manhattan plot for the one-sixteenth distance set-up for the attention-deficit hyperactivity disorder dataset.

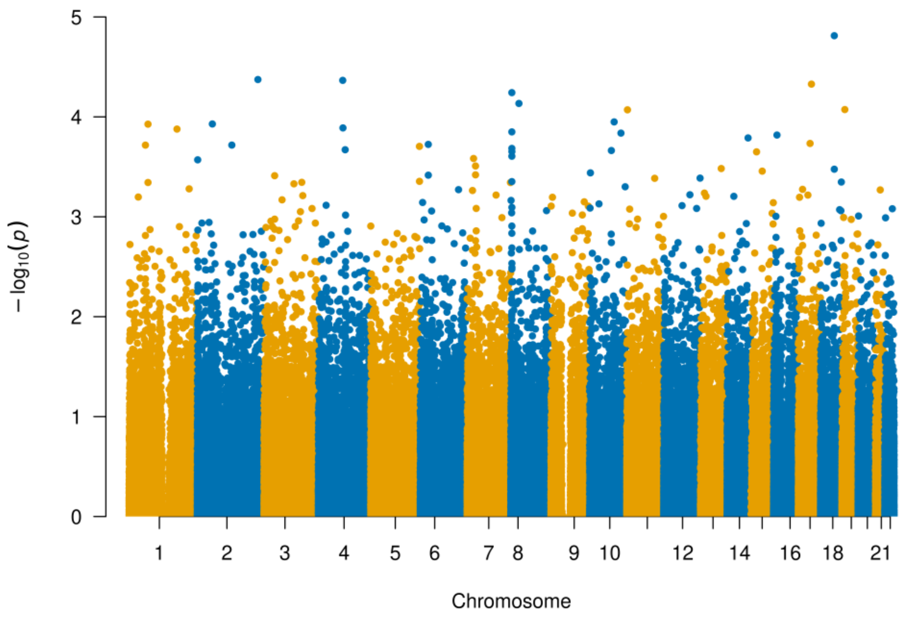

Figure 6. Manhattan plot for the one-sixty fourth distance set-up for the attention-deficit hyperactivity disorder dataset.

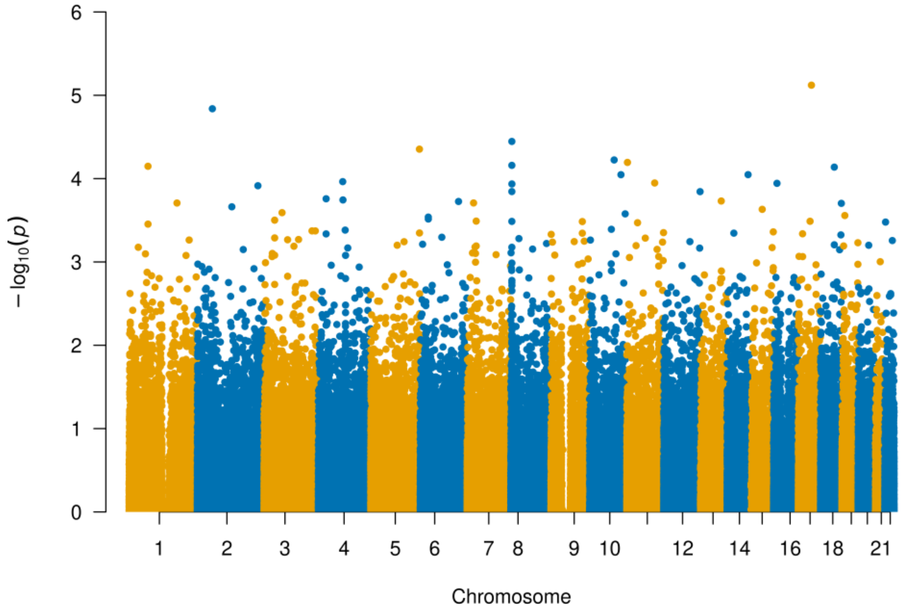

Figure 7. Manhattan plot for the one-one twenty eighth distance set-up for the attention-deficit hyperactivity disorder dataset.

Obsessive Compulsive Disorder Manhattan Plots

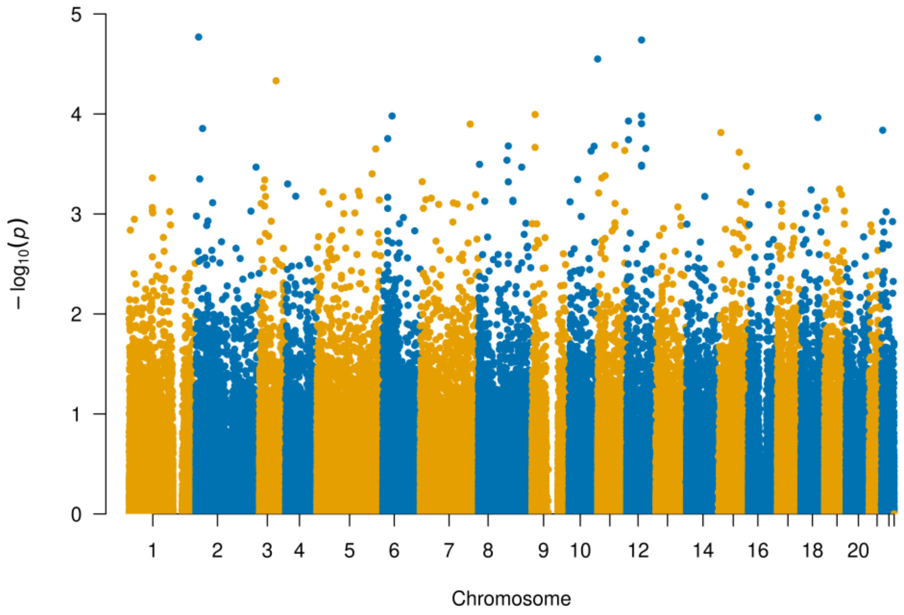

Figure 8. Manhattan plot for the global distance set-up for the obsessive compulsive disorder dataset.

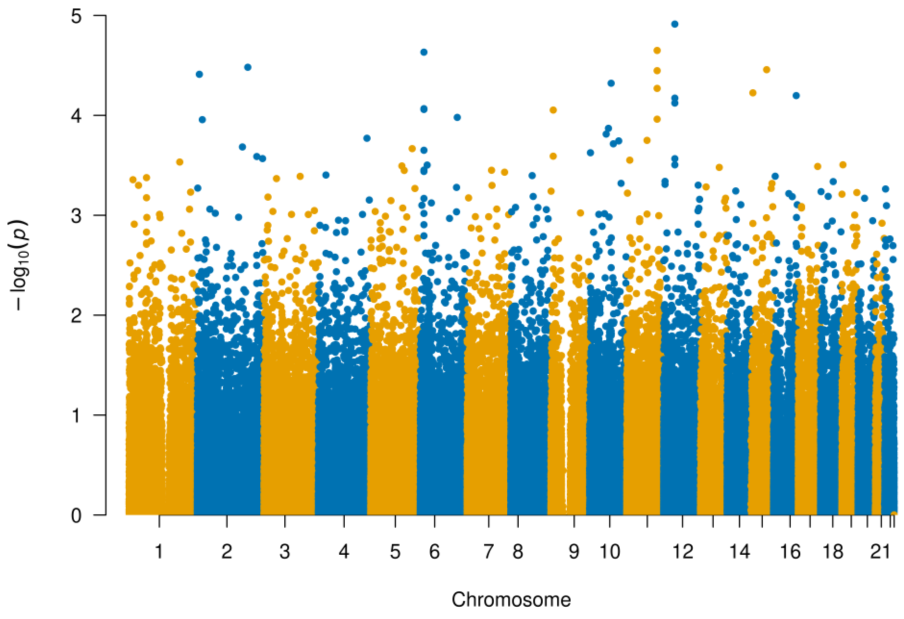

Figure 9. Manhattan plot for the three-quarter distance set-up for the obsessive compulsive disorder dataset.

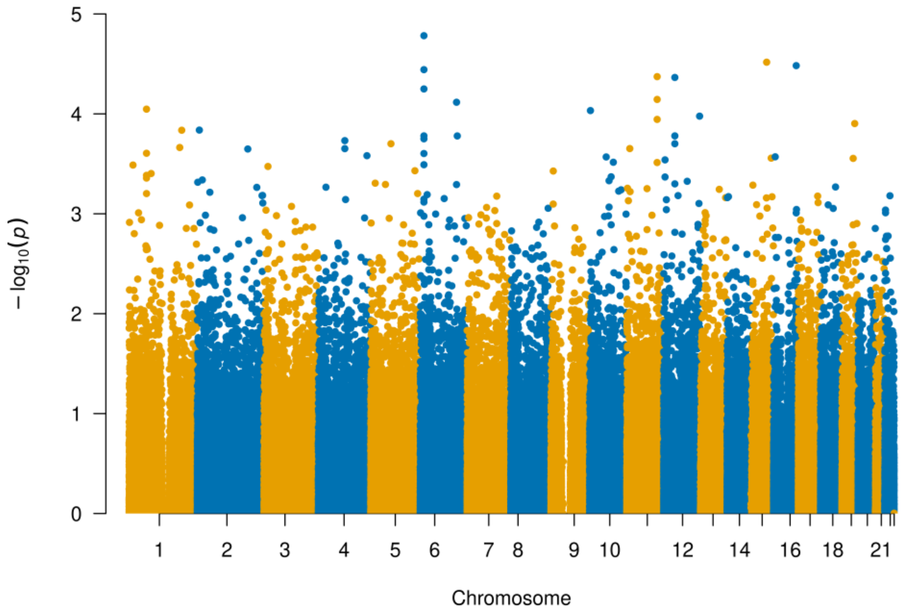

Figure 10. Manhattan plot for the half distance set-up for the obsessive compulsive disorder dataset.

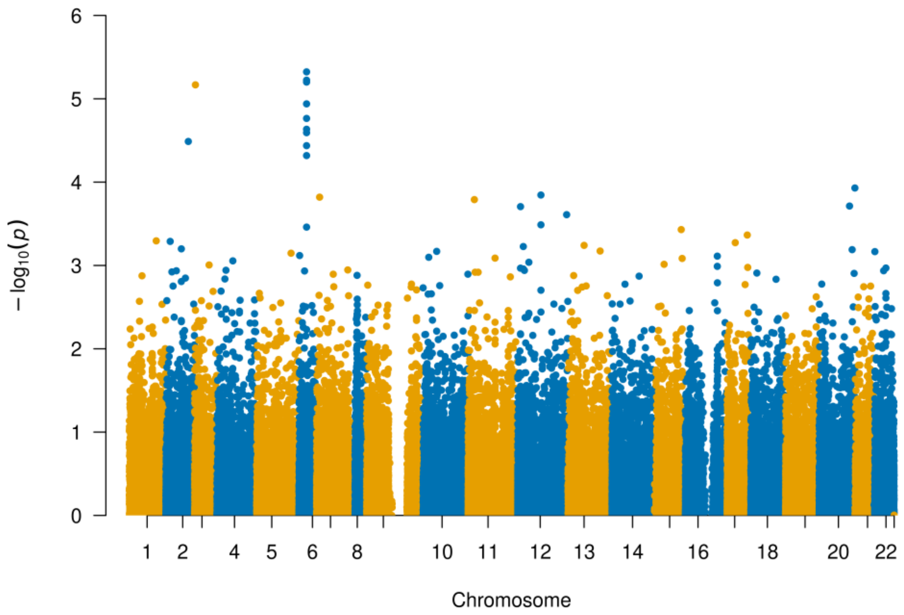

Supplement: Supplementary file 2 [file Data_Sheet_2.pdf]
